# Relationships between leisure participation, leisure constraints, and quality of life among individuals with lower-limb amputations in South Africa

**DOI:** 10.4102/ajod.v14i0.1585

**Published:** 2025-04-29

**Authors:** Adri I. Visser, Mariette Swanepoel, Marike Cockeran, Cindy Kriel

**Affiliations:** 1 Research unit: Physical Activity, Sport and Recreation (PhASRec), Faculty of Health Sciences, North-West University, Potchefstroom, South Africa; 2 Research unit: Medicine Usage in South Africa (MUSA), Faculty Health Sciences, North-West University, Potchefstroom, South Africa

**Keywords:** amputations, leisure participation, leisure constraints, quality of life, prosthetics, lower-limb amputations, TAPES-R, WHOQOL-BREF, WHOQOL-DIS, South Africa

## Abstract

**Background:**

Individuals with lower-limb amputations (LLAs) face unique challenges that affect their leisure participation and overall quality of life (QoL).

**Objectives:**

This study examines the relationships between leisure participation, leisure constraints and QoL among South Africans with LLAs (*N* = 50, mean age 46.2 ± 11.63 years).

**Method:**

A cross-sectional quantitative design was used, collecting data via the Trinity Amputation and Prosthesis Experience Scale-Revised (TAPES-R), Constraints to Participation, the World Health Organization Quality of Life Brief (WHOQOL-BREF), and the World Health Organization Quality of Life Disability Module (WHOQOL-DIS) questionnaires.

**Results:**

Weak to moderate relationships were found between leisure participation and constraints (interpersonal: τb = –0.01, *p* = 0.402; structural: τb = –0.21, *p* = 0.072). Moderate positive associations emerged between leisure participation and QoL in the disability module (τb = 0.21, *p* = 0.073), physical domain (τb = 0.20, *p* = 0.088) and environment domain (τb = 0.20, *p* = 0.091). Medium-negative correlations were observed between QoL and constraints in the physical domain (intrapersonal: *r* = –0.33, *p* = 0.021; interpersonal: *r* = –0.32, *p* = 0.021). Significant negative relationships were found between QoL (social domain) and both intrapersonal (*r* = –0.33, *p* = 0.020) and interpersonal constraints (*r* = –0.36, *p* = 0.010).

**Conclusion:**

This is the first study to explore these relationships in South Africans with LLAs. Intrapersonal and interpersonal constraints significantly impact physical and social QoL. Addressing these barriers may improve overall QoL in this population.

**Contribution:**

This study provides novel insights into the interplay between leisure participation, constraints, and QoL among South Africans with LLAs. By identifying the significant impact of intrapersonal and interpersonal constraints on physical and social QoL, these findings highlight the need for targeted interventions to reduce barriers and enhance leisure engagement.

## Introduction

After sustaining a lower-limb amputation (LLA), individuals often experience a cascade of life-altering changes in various areas of their lives, including on a physical, psychological and social level (Calabrese et al. 2023; Fortington et al. 2013; Razak et al. 2016; Zidarov, Swaine & Gauthier-Gagnon 2009). Various studies indicate a decline in leisure participation, an increase in leisure constraints and a decline in quality of life (QoL) after an LLA (Bragaru et al. 2011; Burger & Marinček 1997; Couture et al. 2010; Ehde et al. 2000; Gallagher et al. 2011; Horgan & MacLachlan 2004; Lee et al. 2022; Kars et al. 2009; Nissen & Newman 1992). Leisure significantly contributes to the QoL (Diaz et al. 2019; Iwasaki 2007). However, individuals living with LLAs often encounter constraints that restrict their participation in leisure activities (Baker & Palmer 2006; Calabrese et al. 2023; Dilworth 2010; Lloyd & Auld 2002).

Leisure refers to time free from life-sustaining responsibilities or work (Henderson 2010; Leitner & Leitner 2012; McLean, Hurd & Rogers 2008). It offers individuals opportunities for self-expression, autonomy, choice, self-definition and relaxation, often lacking in their daily lives (Coleman & Iso-Ahola 1993; Russell 1996). Engaging in leisure activities can yield numerous benefits, such as fostering positive emotions, acquiring knowledge and skills, building social relationships, promoting friendships, relieving stress and enhancing the quality of life (Badia et al. 2013; Calabrese et al. 2023; Creek 2008; Fancourt et al. 2021; Henderson 2010; Kampert & Goreczny 2007; Lloyd & Auld 2002; Melbøe & Ytterhus 2017; Russell & Jamieson 2008; Stumbo & Peterson 2004; Stumbo, Wang & Pegg 2011; Whiting & Hannam 2015). However, as previously noticed, individuals living with LLAs frequently encounter various constraints (intrapersonal, interpersonal and structural) that hinder their participation in leisure activities.

*Leisure constraints* are ‘factors that are assumed by researchers and perceived by individuals to inhibit or prohibit participation in leisure’ (Jackson 2000:62). Crawford, Jackson and Godbey (1991) categorise these constraints into three types: *intrapersonal, interpersonal* and *structural. Intrapersonal constraints* pertain to psychological states, such as stress, anxiety, depression and socialisation challenges during or outside leisure activities (Crawford et al. 1991; Jackson 2000). *Interpersonal constraints* involve relationships and interactions, particularly difficulty finding a suitable partner to engage in leisure activities (Crawford et al. 1991; Jackson 2000). *Structural constraints* include available time, financial resources and the physical environment (Crawford et al., 1991; Jackson 2000). According to Crawford et al. (1991), leisure constraints operate hierarchically and must be overcome sequentially: firstly, *intrapersonal constraints*; secondly, *interpersonal constraints*; and finally, *structural constraints*. Individuals living with LLAs often experience reduced leisure participation, associated with a subsequent decline in or consistently low QoL (Burger & Marinček 1997; Calabrese et al. 2023; Lloyd & Auld 2002).

According to the World Health Organization (WHO 2012), QoL refers to an individual’s perception of their position in life, shaped by their values, culture, goals, expectations and concerns. QoL encompasses various domains, including an individual’s environment, physical and psychological well-being, social relationships, personal beliefs and level of independence (WHO 2012). Albrecht and Devlieger (1999) assert that individuals with a moderate to high QoL are better equipped to manage everyday life and its challenges, thereby contributing to their overall psychological, social and spiritual well-being.

Determining the QoL for individuals living with LLAs can be challenging, particularly when the relationships between *leisure participation, leisure constraints* and *QoL* are not well understood (Wadey & Day 2018). Gallagher et al. (2011) recommend a thorough assessment of the leisure constraints faced by individuals living with LLAs. However, some studies report that individuals with severe disabilities, including LLAs, can still achieve a high QoL (Albrecht & Devlieger 1999; Asano et al. 2008). Although numerous studies have explored the QoL of individuals with LLAs in other countries, there is a significant lack of research in the South African context.

The authors were unable to find existing literature, which has explored the relationships between leisure participation, leisure constraints and QoL in individuals living with LLAs. Addressing this gap will contribute valuable insights to the global and national scientific knowledge base. While the findings of this study are specific to South Africa, they could serve as a basis for comparison in other contexts. This study aims to explore the relationships between leisure participation, leisure constraints and quality of life among individuals living with LLAs in South Africa.

## Research methods and design

### Study design

The research design employed in this study was a cross-sectional quantitative approach that utilised an online survey. Both availability and snowball sampling techniques were utilised to collect data comprehensively and inclusively.

### Setting

Mediators from both public and private sectors in South Africa who had direct contact with individuals living with LLAs distributed email invitations to potential participants.

### Study population and sampling strategy

Given the limited number of LLA individuals in South Africa, a minimum sample size of 42 participants was sought to ensure an acceptable reliability coefficient (*r* > 0.6) with a significance level of α = 0.05 and β = 0.20 (Resnik & Borgia 2011). A total of 50 individuals participated (68% male, 30% female, 2% preferred not to specify). A convenience sample of South African individuals aged 18 years or older (mean age = 46.2 years) with LLAs was invited to participate in this study. Inclusion criteria required participants to have had an LLA for at least 18 months, be able to read and understand English, and have access to email and the internet. The exclusion criteria were those who were pregnant during the study or had other disabilities or injuries apart from their LLA.

Participants willing to participate in the study were asked to complete an online survey using Google Forms. The initial page of the survey included a study description and an informed consent document. Participants gave their consent by clicking a checkbox, which granted them access to the survey questions. Initially, 46 surveys were submitted. Four additional participants completed the survey through snowball sampling, resulting in a total of 50 submissions, all of which were included in the analysis.

### Data collection

Data were collected using an online Google survey, including demographic information from the TAPES-R, Constraints to Participation and WHOQOL-BREF and WHOQOL-DIS questionnaires.

Leisure constraints were determined using the ‘Constraints to Participation’ questionnaire designed for individuals with mental and physical disabilities. It was employed in this study to evaluate the participation in leisure activities and the constraints faced by individuals living with LLAs, as detailed in Table 1a and Table 1b. The reliability of the questionnaire was confirmed using Cronbach’s alpha. The results were as follows: community (α = 0.92), time (α = 0.84), equipment (α = 0.87), economic (α = 0.90), intrapersonal (α = 0.81), transport (α = 0.80) and interpersonal (α = 0.84), as indicated by Darcy, Lock and Taylor (2017). Leisure participation was classified into three categories: low (no participation and once per month, once per month), moderate (once per week) and high (more than once per week or daily). The original questionnaire had seven constraint sections: community, time, equipment, economics, intrapersonal, transport and interpersonal. However, for this study, the questions were organised into three categories, as suggested by Crawford et al. (1991): intrapersonal, interpersonal and structural constraints. Responses were documented using a 6-point Likert scale ranging from 1 – 6 (1 = Never, 2 = A little, 3 = Sometimes, 4 = Often, 5 = Most of the time, 6 = Always).

**TABLE 1a:** Demographic information and descriptive statistics of the TAPES-R questionnaire, leisure participation, leisure constraints and quality of life (*N* = 50).

Variable	%
**Gender**	
Males	68
Females	30
Prefer not to say	2
**Type of amputation**	
Below the knee	62
Above the knee	34
Another type (through-knee)	4
**Reason for the amputation**	
Accident related	52
Other†	28
Diabetes	10
Peripheral vascular disease	6
Cancer	4
**Leisure participation**	
Low (no participation or once per month)	20
Moderate (once per week)	16
High (more than once per week, or daily)	56

Note: Age = 46.22 (±11.63); Duration individuals have had their amputation (months) = 120.88 (±120.18).

TAPES-R, Trinity Amputation and Prosthesis Experience Scale-Revised.

† , armed robbery, blood clots, pressure sores, congenital deformities, sports injuries, fibular hemimelia, infections and septicaemia.

**TABLE 1b:** Demographic information and descriptive statistics of the TAPES-R questionnaire, leisure participation, leisure constraints and quality of life (*N* = 50).

Variable	Mean	SD	Min.	Max.	25^th^ percentile	50^th^ percentile	75^th^ percentile
**Leisure constraints†**							
**Constraint area**							
Intrapersonal	1.89	0.38	1.00	3.46	1.31	1.65	2.10
Interpersonal	1.88	0.22	1.00	5.00	1.00	1.50	2.38
Structural	2.22	0.46	1.00	4.45	1.59	2.38	2.69
**Quality of life domain scores of the WHOQOL-BREF and WHOQOL-DIS questionnaire‡**							
**Domain areas**							
Disability module	79.46	15.02	37.50	100.00	70.31	82.29	90.10
Psychological	77.40	20.58	25.00	100.00	63.75	80.00	95.00
Physical	73.00	20.15	17.86	100.00	59.82	76.79	89.29
Social	78.50	18.68	33.33	100.00	66.67	79.17	93.75
Environment	73.33	19.58	29.17	100.00	58.33	77.08	91.67

Note: WHOQOL-DIS (World Health Organization Quality of Life Disabilities Module) is an instrument designed to assess the quality of life for individuals with intellectual and physical disabilities and specifically evaluates their experiences and well-being. WHOQOL-BREF (World Health Organization Quality of Life Brief) is a shorter 26-item version of the instrument.

Min., minimum; Max., maximum; SD, standard deviation; TAPES-R, Trinity Amputation and Prosthesis Experience Scale-Revised.

† , Likert scale values: 1 = Never; 2 = A little; 3 = Sometimes; 4 = Often; 5 = Most of the time; 6 = Always;

‡ , 0 = lowest score; 100 = highest score.

Quality of life was determined using the WHOQOL-BREF and WHOQOL-DIS questionnaire, which comprises five domains: the disability module, psychological, physical, social and environmental. These domains were specifically developed to measure the QoL of individuals with physical or intellectual disabilities. This study used the WHOQOL-BREF and WHOQOL-DIS questionnaire to assess QoL among individuals with physical disabilities. The overall Cronbach’s alpha for the WHOQOL-DIS module was 0.852, with all corrected item-total correlation values exceeding 0.35 (ranging from 0.373 to 0.613) (Power, Green & WHOQOL-DIS group 2010). The questionnaire includes 36 questions, three general questions and the remaining assessing the five domain scores. Responses were collected using a 5-point Likert scale, where 1 = Not at all, 2 = A little, 3 = Moderately, 4 = Mostly and 5 = Totally. The WHOQOL-BREF and WHOQOL-DIS questionnaire contains four negatively worded, reverse-scored questions. Each domain score was scaled positively, meaning higher scores indicated a higher QoL. Domain scores were calculated by determining the mean score for each item and multiplying these mean scores by four to make them comparable with those used in the WHOQOL-100 (WHO 2012). The WHOQOL-BREF and WHOQOL-DIS questionnaire scores were reported on a 0–100 scale for each domain.

### Data analysis

Data analyses were performed using SPSS version 27. Descriptive statistics, including mean values, standard deviations, minimum and maximum values, frequencies and percentiles, were computed for the Constraints to Participation questionnaire and the WHOQOL-BREF and WHOQOL-DIS questionnaire. Spearman’s rank correlation and Kendall’s tau-b were employed to assess the relationships between variables. Statistical significance was set at *p* < 0.05.

### Ethical considerations

Ethical approval was obtained from the NWU Health Research Ethics Committee of the NWU (HREC) (reference: NWU-00034-16-A1). Prior to completing the survey, mediators explained the study’s purpose and procedures, emphasising that participation was voluntary and participants could withdraw at any time without consequence. No physical risks were involved, and minimal emotional discomfort was anticipated, as the survey did not address sensitive topics. Confidentiality was ensured, and participants were informed of the potential for online data security breaches.

## Results

The study sample comprised 50 participants with an average age of 46.22 years (±11.63), including 68% males, 30% females, and 2% who preferred not to disclose their gender. The average duration since amputation was 120.88 months (±120.18). The majority of participants had below-the-knee amputations (62%), followed by above-the-knee amputations (34%) and other types (through-knee) at 4%. The primary reasons for amputation were accident-related (52%), followed by other causes such as armed robbery, blood clots, pressure sores, congenital deformities, sports injuries, fibular hemimelia, infections and septicaemia (28%). Diabetes accounted for 10% of cases, peripheral vascular disease for 6%, and cancer for 4%.

In terms of leisure participation, 56% of participants engaged in activities at a high frequency (once per week or daily), 16% participated moderately (once per week), and 20% reported low participation (no participation or once per month or less). Leisure constraints were assessed using the previously mentioned 6-point Likert scale, with intrapersonal and interpersonal constraints showing minimal impact (mean values of 1.89 and 1.88, respectively), while structural constraints were slightly higher (mean value of 2.22).

Quality of life scores across various domains, as measured by the WHOQOL-BREF and WHOQOL-DIS questionnaire, indicated generally high levels of well-being. Scores for the disability module averaged 79.46 (SD = 15.02), psychological domain scores averaged 77.40 (SD = 20.58), physical domain scores averaged 73.00 (SD = 20.15), social domain scores averaged 78.50 (SD = 18.68), and environment domain scores averaged 73.33 (SD = 19.58). These findings reflect a diverse range of experiences among individuals with LLAs, with varying levels of leisure participation and constraints and high QoL scores across different domains.

The analysis of the relationship between leisure participation and leisure constraints (Table 2) revealed varying degrees of correlation across different constraint areas. A very weak, non-significant correlation was found between *leisure participation* and *intrapersonal constraints* (τb = −0.01, *p* = 0.992). Similarly, the relationship between *leisure participation* and *interpersonal constraints* was weak and non-significant (τb = 0.01, *p* = 0.402). However, a moderate negative correlation was observed between *leisure participation* and *structural constraints* (τb = −0.21), approaching significance (*p* = 0.072). These findings suggest that structural constraints may moderate leisure participation, whereas intrapersonal and interpersonal constraints show negligible influence.

**TABLE 2:** Relationships between leisure participation and leisure constraints.

Kendall’s tau-b correlation: Constraint areas	Leisure participation	
	Correlation coefficient (𝝉b)	*p*-value Sig. (2-tailed)
Intrapersonal	−0.01	0.992
Interpersonal	0.010	0.402
Structural	−0.21	0.072

Note: 𝝉b = Kendall’s tau-b correlation coefficient values: 0.10 to 0.19 = weak; 0.20 to 0.29 = moderate; 0.30 or above = strong; Sig., significant.

Table 3 presents the relationships between *leisure participation* and *QoL*. Although the results were not statistically significant, moderate positive correlations were observed between *leisure participation* and the *disability module* (τb = 0.21; *p* = 0.073), *physical domain* (τb = 0.20; *p* = 0.088) and *environment domain* (τb = 0.20; *p* = 0.091) of the WHOQOL-BREF and WHOQOL-DIS questionnaire. These findings suggest a potential association between increased leisure participation and improved QoL across various domains, although the relationships did not reach statistical significance.

**TABLE 3:** Relationships between leisure participation and quality of life among individuals with lower-limb amputations.

Kendall’s tau-b correlation: WHOQOL-BREF and WHOQOL-DIS questionnaire domains	Leisure participation	
	Correlation coefficient (τb)	*p*-value Sig. (2-tailed)
Disability module	0.21	0.073
Physical	0.20	0.088
Psychological	0.05	0.698
Social	−0.04	0.754
Environment	0.20	0.091

Note: 𝝉b = Kendall’s tau-b corrélations coefficient values: 0.10 to 0.19 = weak; 0.20 to 0.29 = moderate; 0.30 or above = strong.

LLAs, lower-limb amputations; WHOQOL-BREF, World Health Organization Quality of Life Brief; WHOQOL-DIS, World Health Organization Quality of Life Disability Module.

The analysis revealed several significant relationships between *leisure constraints* and *QoL* (Table 4) among individuals with LLAs. Specifically, there were moderate negative correlations between *intrapersonal constraints* and both the *physical* (*r* = −0.33, *p* = 0.021) and *social domains* (*r* = −0.33, *p* = 0.020) of QoL. *Interpersonal constraints* also showed a moderate negative correlation with the *physical* (*r* = −0.32, *p* = 0.021) and *social domains* (*r* = −0.36, *p* = 0.010). However, no significant correlations were found between *structural constraints* and the *QoL domains*, although a trend was observed with the *physical domain* (*r* = −0.26, *p* = 0.065). The other domains and the disability module did not correlate significantly with leisure constraints. These findings suggest that *intrapersonal constraints* and *interpersonal constraints* notably impact the *physical* and *social aspects* of *QoL* for individuals with LLAs, highlighting the importance of addressing these barriers to improve overall well-being.

**TABLE 4:** Relationships between leisure constraints and quality of life of individuals with lower-limb amputations.

Spearman’s rho correlations: WHOQOL-BREF and WHOQOL-DIS questionnaire domains	Leisure constraints					
	Intrapersonal		Interpersonal		Structural	
	*r*-value	*p*-value	*r*-value	*p*-value	*r*-value	*p*-value
Physical	−0.33*	0.021	−0.32*	0.021	−0.26	0.065
Psychological	−0.08	0.600	−0.04	0.760	−0.21	0.144
Social	−0.33*	0.020	−0.36**	0.010	−0.24	0.100
Environment	−0.12	0.418	−0.02	0.869	−0.15	0.289
Disability module	−0.20	0.161	−0.18	0.191	−0.18	0.206

^Note:^ *r*^-values: 0.1 = small relationship; 0.3 = moderate relationship and 0.5 = strong relationship.^

* , Correlation is significant at the 0.05 level (2-tailed);

** , Correlation is significant at the 0.01 level (2-tailed).

## Discussion

This cohort exhibited high levels of *leisure participation*, generally low *leisure constraints*, and elevated *QoL* scores across all domains (Table 1a and b). Most participants engaged in leisure activities regularly, reporting frequent participation. *Leisure constraints* were consistently low across intrapersonal, interpersonal and structural categories, while QoL scores were notably high in domains such as disability, psychological and social. These findings contrast with several studies that report a decline in leisure participation, increased leisure constraints and lower QoL scores among individuals with LLAs (Bragaru et al. 2011; Calabrese et al. 2023; Couture et al. 2010; Ehde et al. 2000; Gallagher et al. 2011; Kars et al. 2009; Nissen & Newman 1992; Westlund 2021; Sinha, Van Den Heuvel & Arokiasamy 2011). Matos, Naves and De Araujo (2020) suggested that the time since amputation and the type of amputation significantly influence an individual’s adjustment and overall QoL. In this cohort, participants had lived with their amputations for an average of 10 years, with 62% having below-knee LLAs, which may have facilitated their adjustment and contributed to their high QoL. Jackson (1999) posits that individuals can adapt their behaviour to maintain leisure involvement by negotiating constraints through various strategies. This suggests that the participants in this study may have effectively ‘overcome’ their leisure constraints.

Understanding the relationship between *leisure participation* and *leisure constraints* is essential for examining the lived experiences of individuals with LLAs. This study identified two noteworthy negative correlations that provide insights into this dynamic (Table 2). Firstly there was a weak negative correlation between *leisure participation* and *interpersonal constraints*. This finding suggests that participants may have benefitted from robust social networks, including friends and family, who likely facilitated and encouraged their engagement in leisure activities. This aligns with previous research by Bragaru et al. (2011) and Olsen et al. (2022), which underscored the pivotal role of social support in promoting leisure participation among individuals with LLAs. Secondly there was a moderate negative correlation between *leisure participation* and *structural constraints*. This highlights the potential influence of reduced physical or environmental barriers on enabling greater involvement in leisure pursuits. Similar results were reported by Day, Wadey and Strike (2019), who observed that individuals with LLAs were more likely to engage in activities such as walking, running, socialising and football on days when physical discomfort, including phantom limb pain, pressure sores or swelling, was minimal.

While examining the relationships between *leisure participation* and *QoL* domains (Table 3), several moderate positive correlations, although not statistically significant, were identified between *leisure participation* and the following *QoL* domains: the *disability domain*, the *physical domain*, and the *environment domain*. These correlations indicate that engagement in leisure activities may positively impact QoL. This finding is consistent with Lyu, Oh and Lee (2013), who observed that individuals often adapt their circumstances to engage in preferred leisure activities. It also aligns with Jackson’s (1999) theory, which highlights how individuals negotiate and overcome barriers to achieve their leisure goals. Moreover, the results support Albrecht and Devlieger’s (1999) assertion that individuals with long-lasting limitations can attain a high QoL despite the challenges they face.

The analysis of the relationship between *leisure constraints* and *QoL* (Table 4) provides valuable insights, highlighting moderate negative correlations between the *physical domain* and both *intrapersonal constraints* and *interpersonal constraints*. These findings align with previous results, which indicated high physical domain scores and low constraint levels within our cohort. One potential explanation for these moderate negative relationships is that physical activities and physical health were highly valued by participants, who often engaged in such activities with friends. Dean, McFadyen and Rowe (2008) support this explanation, suggesting that participation in physical activities enhances physical health and fosters social connections.

Table 4 also reveals a significant moderate negative relationship between the *social domain* of the WHOQOL-BREF and WHOQOL-DIS questionnaire and *intrapersonal constraints*. This suggests that participants valued their engagement in community social leisure activities and did not experience significant anxiety or stress related to these activities. Godbey (2003) observes that intrapersonal constraints might include a desire to participate in leisure activities despite feeling depressed; however, our cohort seemed free from such constraints, as evidenced by their active involvement in community activities. A significant moderate negative relationship was also observed between the *social domain* and *interpersonal constraints*. This indicates that our cohort’s low level of interpersonal constraints facilitated their social interactions, allowing them to integrate well with others and meet their social needs, supported by peers (Bragaru et al. 2011; Dean et al. 2008 & Olsen et al. 2022). Overall, the cohort reported low scores for all constraints, with *structural constraints* having a slightly higher mean value. These findings confirm that participants have effectively adapted to their LLA and enjoy a high quality of life while encountering minimal leisure constraints. The results underscore the importance of reducing leisure constraints to enhance participation in activities for individuals with LLAs.

## Conclusion

Our research represents the first study to explore the relationship between leisure participation, leisure constraints and QoL among individuals living with LLAs in South Africa. We identified a weak to moderate negative relationship between leisure participation and certain leisure constraints, specifically interpersonal and structural constraints. This suggests that higher leisure participation is associated with fewer interpersonal and structural constraints. When examining the link between leisure participation and QoL, we found non-significant moderate positive correlations with the disability module, physical domain and environment domain of the WHOQOL-BREF and WHOQOL-DIS questionnaire. This indicates that greater leisure participation is associated with higher QoL scores in these domains. In addition, significant moderate negative correlations were observed between leisure constraints (both intrapersonal and interpersonal) and QoL, particularly in the physical and social domains. These results suggest that increased intrapersonal and interpersonal constraints are linked to decreased QoL in these areas.

## Recommendations

To gain a deeper understanding of how LLA affects participation in leisure activities, conducting interviews and focus groups with participants could be highly informative. Interviews can reveal whether there was a decline in leisure participation following the amputation and explore whether individuals were more active in leisure activities before the procedure. This approach provides a comprehensive view of the impact of LLAs on leisure engagement and helps to identify the reasons behind changes in activity levels.

While questionnaires offer valuable insights, interviews with individuals with physical disabilities can provide more nuanced feedback. This method allows for clarification of responses and exploration of detailed experiences. For instance, asking questions such as, ‘How did your engagement in leisure activities change before and after your lower limb amputation?’ can yield richer data than questionnaires, which may not capture all relevant information.

The Constraints to Participation questionnaire highlighted that intrapersonal constraints were barriers to leisure activity engagement. Participants mentioned involvement in other leisure activities, suggesting a need for further investigation into their activities. Future research should focus on identifying these ‘other’ activities to understand better the leisure pursuits of individuals with LLAs and the nature of intrapersonal constraints affecting their participation.
